# A dedicated mental resource for intuitive physics

**DOI:** 10.1016/j.isci.2023.108607

**Published:** 2023-12-01

**Authors:** Alex Mitko, Ana Navarro-Cebrián, Sarah Cormiea, Jason Fischer

**Affiliations:** 1 Department of Psychological and Brain Sciences, Johns Hopkins University, Baltimore, MD, USA; 2 Department of Psychology, University of Maryland, College Park, MD, USA; 3 Department of Neurology, University of Pennsylvania, Philadelphia, PA, USA

**Keywords:** Physics, Neuroscience, Cognitive neuroscience

## Abstract

Countless decisions and actions in daily life draw on a mental model of the physical structure and dynamics of the world – from stepping carefully around a patch of slippery pavement to stacking delicate produce in a shopping basket. People can make fast and accurate inferences about how physical interactions will unfold, but it remains unclear whether we do so by applying a general set of physical principles across scenarios, or instead by reasoning about the physics of individual scenarios in an ad-hoc fashion. Here, we hypothesized that humans possess a dedicated and flexible mental resource for physical inference, and we tested for such a resource using a battery of fine-tuned tasks to capture individual differences in performance. Despite varying scene contents across tasks, we found that performance was highly correlated among them and well-explained by a unitary intuitive physics resource, distinct from other facets of cognition such as spatial reasoning and working memory.

## Highlights

• A common mental resource contributes to performance in a range of physical scenarios
• Physical reasoning is separable from other cognitive domains, e.g., spatial cognition
• The test of intuitive physics is a reliable measure of physical reasoning ability

## Graphical abstract

**Figure:**
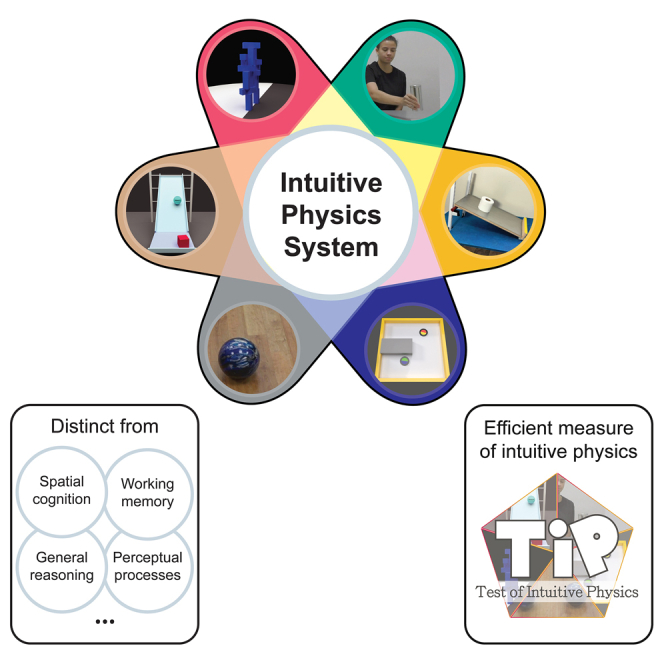

## Introduction

The activities of daily life continually draw on our ability to apprehend the physical structure of the world and predict how it will behave in the coming moments. When emptying the dishwasher, we must judge which stacking arrangements of cups and plates will be stable and leave the items on the bottom unharmed. When a mishandled clementine rolls across the counter, we have a split second to judge whether its speed and trajectory will carry it off the counter’s edge, and we must precisely predict its path as it falls in order to catch it. We carry out these judgments with apparent ease despite the vast diversity of physical structure and dynamics in the scenarios that we regularly encounter. How do we form these inferences?

**Figure 1:**
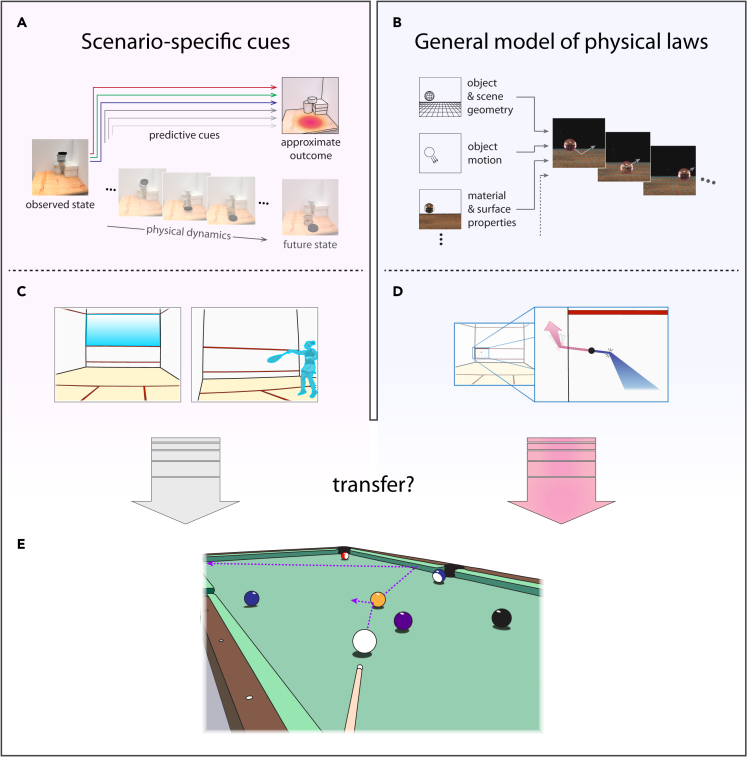
Two approaches to physical prediction (A) Cue-based prediction leverages features of the scene that have proven informative about physical outcomes in previous experiences. Informative cues collectively point toward more likely outcomes without the need to predict the precise dynamics leading to the final state. (B) Prediction based on a mental model of the physical laws governing a scene’s behavior. Using estimates of relevant scene variables, this approach applies physical laws to anticipate how interactions will play out from one moment to the next, arriving at a prediction of the dynamics leading to the end state of interest. (C) Cue-based physical prediction in a squash match. A player might use cues such as the location of a bounce relative to visual landmarks or the body posture of their opponent to anticipate where the ball will arrive without the need to trace its predicted path. (D) Applying physical laws in a squash match. A player might use the observed speed and direction of the ball, along with knowledge about the ball’s soft material and the smoothness of the walls, to predict the path that the ball will take as it ricochets on its return. (E) Transfer of physical prediction approaches across scenarios. While the same physical laws can be applied to predict the behaviors of a squash ball and a billiard ball, some predictive cues from the squash match will be absent or uninformative in a game of pool.

Figure 1 illustrates two potential accounts of the mental processes underlying our ability to form rapid, online predictions about how physical events will unfold – an ability termed *intuitive physics*. One approach (Figure 1A) leverages scenario-specific cues that have been learned on the basis of their reliability in predicting the outcomes of similar past experiences. These cues need not have their basis in the laws of physics so long as they serve as reliable predictors of the outcomes we care about. Figure 1C depicts a concrete example: imagine that a friend challenges you to a game of squash. Even if this is your first time on the court, you might quickly pick up on some useful cues for predicting where the ball will travel – for example, perhaps you notice that when your friend’s shot bounces above the service line painted on the back wall, the ball tends to arrive high over your head on its return. Picking up on a collection of these cues, which might relate to things like the order in which the ball contacts multiple walls or even the position where your friend is standing, could help you develop an accurate sense of where the ball will arrive after each volley. In a more general sense, this kind of cue-based strategy could be a successful one in a variety of contexts – associating specific cues with particular physical outcomes in your everyday activities could help you behave effectively in the range of scenarios that you regularly encounter.

At the same time, relying solely on scenario-specific cues would fail to take advantage of the fact that a common set of underlying physical laws governs the behaviors of all scenarios you encounter, including those you’ve never experienced before. A mental system that used the underlying causal structure of a scene as its basis for prediction (Figure 1B) would be effective even when shortcuts to inferring physical outcomes aren’t available. In the example of the squash match, an alternative to cue-based prediction (though not mutually exclusive) would be to estimate how the ball will travel as it arcs toward the ground due to gravity, using your knowledge of its soft rubber material to predict the angle it will take as it bounces off the walls and heads in your direction (Figure 1D). Under this approach, the relevant information for prediction lies in the physical variables that determine how events will unfold – velocity, mass, surface friction, and shape, to name a few. The prediction algorithm is a mental model – however rudimentary – of the way in which objects behave and interact under the laws of physics. Physical laws could be applied to predict dynamics via simulation, as proposed by Battaglia, Hamrick, and Tenenbaum.^1^ Other approaches such as rule-based reasoning could in principle function to apply physical laws as well, so long as they capture the underlying principles governing physical behaviors rather than more superficial predictive cues (see discussion).

The two accounts in Figure 1 are thus distinguished both by the nature of the information they operate on (predictive cues vs. physical contents of the scene) and by the way in which they arrive at a predicted outcome, either bypassing the particulars of how physical events play out or endeavoring to model those events as a means of arriving at the outcome in question. The two approaches also differ in a third key respect: the degree to which their successful application in one scenario will transfer to other contexts involving different objects and scene configurations. Returning to the example of the squash match a final time, imagine that after the vigorous match where you held your own, the two of you decide to wind down with a game of pool. Will your impressive performance on the squash court extend to the pool hall? If your predictions of the squash ball’s behavior were based on cues involving the markings on the court, your friend’s body posture, or even the particular degree to which the ball lost speed after a bounce, those cues will be of no use at the pool table – you will need to learn a new set of cues for the new scenario. If, on the other hand, you succeeded by predicting how the ball would travel based on the underlying physical principles governing its behavior, then you need only to update the variables in your mental model to match those of the new context – the billiard balls are harder, heavier, and smoother than the squash ball, while the bumpers on the table are softer than the walls surrounding the court. An intuitive physics system that models the underlying causal structure of a scene as its basis for prediction has the advantage of generalizing across a variety of physical scenarios even when the scenarios share few of their surface-level features.

In this study, we tested for the existence of a general physical prediction resource in the human mind by looking for its signature in the transfer of performance across tasks. It is critical to note that the two different perspectives on how physical predictions are generated – via cue-based inference or via a model of underlying physical laws – are not incompatible. It is surely the case that people learn the predictive cues in scenarios where they have repeated experience, and they leverage those cues to improve the efficiency and precision of their physical inferences. Examples of such cases have been demonstrated both for commonplace tasks like reasoning about containers^2^ and for highly trained tasks like professional sports.^3^^,^^4^^,^^5^ The question at hand, then, is whether this cue-related information sits on the foundation of a more general and flexible mental model of the underlying causal structure common to all scenarios we encounter.

A flexible mental resource capable of supporting physical inference across a variety of scenarios would not necessarily need to have its functions constrained to the domain of intuitive physics (for example, performance could be supported by multiple demand resources that contribute to performance on a wide range of tasks [^6^]). Our hypothesis here, however, is that the flexibility of a mental resource supporting intuitive physics would derive from the application of physical laws to infer physical behaviors. Under this account, the intuitive physics system would be both general in its capacity to operate over a broad range of physical scenarios and domain-specific for the processing of scene structure and behaviors that can be modeled with Newtonian mechanics. Our experiments here will test for both the flexibility of the mental processes underlying physical prediction and their specificity to the domain of intuitive physics.

Here, we use an individual differences approach to test for the existence of a flexible physical prediction resource that contributes to performance across a wide variety of physical tasks. We characterize intuitive physics performance on a collection of tasks designed to differ markedly in their visuospatial contents and particular inference demands, and test for an underlying factor that drives correlated performance across tasks. A crucial aspect of this endeavor is the careful fine-tuning of each task to capture reliable individual differences in performance. Our resulting task battery is calibrated to the range of performance across typical individuals and enables a view of how intuitive physics performance in a given scenario translates to other scenarios. Note that this work does not assume that *general* implies *homogeneous*. Here, we conceive of a general physical prediction resource as a collection of inference mechanisms (i.e., simulation, rule based, hybrid, or other) that collectively support physical prediction ability across a wide range of scenarios. It is also important to note that *general* (i.e., recruited for a variety of intuitive physics tasks) does not necessarily imply *specialized*. In the work here, we also compare intuitive physics performance with other tasks that measure related abilities and have been shown to track closely with general intelligence as well – namely, working memory and spatial cognition. To test intuitive physics abilities within a shorter time frame, we constructed a condensed version of the physics task battery (the Test of Intuitive Physics; TIP). The TIP provides an efficient means of measuring general intuitive physics abilities in a variety of participant populations and experimental contexts going forward.

To anticipate, our findings reveal tightly correlated individual differences in performance across a collection of tasks whose key commonality is physical inference. These individual differences cannot be accounted for by the other facets of cognition we tested (spatial cognition, working memory, and other general sources of variation in performance), pointing to a flexible and dedicated mental resource for intuitive physics.

## Results

Our overarching aims were two-fold: first, to test for evidence of a dedicated mental resource for intuitive physics that supports physical inferences across a variety of physical scenarios, and second, in the case that we found compelling evidence for such a resource, to develop a compact and efficient behavioral measure that can be employed to test general intuitive physics ability in future work. A challenge in addressing each of these objectives is that little prior work has been specifically focused on developing and validating intuitive physics tasks that are sensitive to individual differences in performance. To tackle that challenge, we first set out to assemble a battery of intuitive tasks with two key properties. First, we designed the task battery to have substantial differences in scene contents and inference demands across tasks. A collection of tasks that are too similar in their contents would not provide a good test of generalization across scenarios. To return to the squash example depicted in Figure 1, if a player was placed in a “new” scenario that involved increasing the dimensions of the squash court, many of the informative situation-specific cues would still apply. We aimed to generate a set of tasks in which both the scene structure and the type of inference (e.g., predicting a future state or inferring a latent physical property) varied substantially across tasks. At the same time, we maintained some key commonalities across tasks such as the depiction of the scenarios from the observer’s first-person perspective within their personal interaction space, which may be critical to engaging the intuitive physics system in its native coordinate frame.^7^^,^^8^ Our second priority when generating tasks was to calibrate each one to the dynamic range of performance across individuals. We fine-tuned the difficulty of each task so that typical adults would vary in their performance from near chance in the lowest performers to near ceiling in the best performers, allowing us to measure reliable individual differences. We achieved this goal by way of extensive piloting for each task, selecting trials that reliably captured individual differences (see STAR Methods).

**Figure 2:**
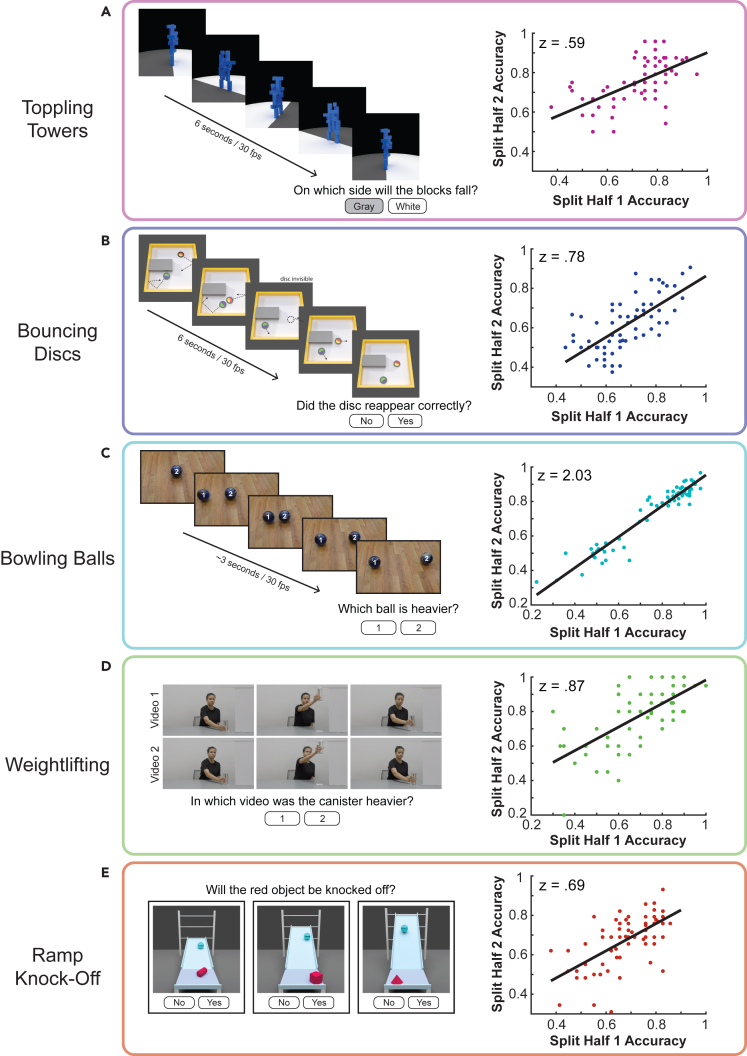
Battery of five intuitive physics tasks used in this study In each panel, an example trial is shown on the left, and the split half reliability of the task is shown in a scatterplot on the right, computed by randomly splitting each participant’s data in half and computing the correlation between performance in the two independent halves of the data (see STAR Methods). (A) Toppling Towers task: participants saw a 360-degree camera pan of an unstable block tower and reported the side of the supporting platform on which the majority of the blocks would come to rest after the tower collapsed. Split-half reliability was z = 0.59, p < 0.001, N = 71. (B) Bouncing Discs task: on each trial, participants saw a video of two discs gliding within an arena, bouncing off of walls, barriers, and each other. After 2 s, one of the discs disappeared from view but continued to physically interact within the scene. The disc remained invisible for 2 s and then reappeared and was visible for the final 2 s of the video. Participants judged whether the hidden disc reappeared at the correct location and with the correct velocity, requiring them to mentally track the disc’s behavior while it was hidden. Split-half reliability was z = 0.78, p < 0.001, N = 71. (C) Bowling Balls task: in each trial, participants viewed a collision between two bowling balls of different weights. One ball began in a static position in the center of the scene and another ball entered from the left, striking and launching the static ball. Participants judged which ball was heavier based on the observed collision. Split-half reliability was z = 2.03, p < 0.001, N = 71. (D) Weightlifting task: participants saw two videos of a person lifting a nondescript metal canister, with the two videos presented in sequence. Participants judged which canister was heavier based on the observed lifts. Split-half reliability was z = 0.87, p < 0.001, N = 71. (E) Ramp Knock-off task: in each trial, participants saw a static scene in which a ball was positioned at the top of a sloped ramp and another object was resting on a platform at the bottom. Across trials, the weight of the ball, slope of the ramp, shape of the red object at the bottom, and positions of both the ball and the red object varied. Participants judged whether the ball would knock the red object off the lower platform when it was released. Successful performance required jointly considering all the aforementioned variables. Split-half reliability was z = 0.69, p < 0.001, N = 69.

The resulting collection of tasks is shown in Figure 2. The *Toppling Towers* task Figure 2A) required participants to judge the direction that an unstable block tower would fall, the *Bouncing Discs* task (Figure 2B) required participants to predict the trajectory and spin of a puck over a period of time in which it was hidden from view, the *Bowling Balls* task (Figure 2C) required participants to infer the relative weights of two bowling balls after seeing them collide, the *Weightlifting* task (Figure 2D) required participants to infer the weight of a nondescript canister after seeing a person lift it, and the *Ramp Knock-Off* task (Figure 2E) required participants to predict whether an object would be knocked off a platform by a ball that strikes it. Several of these tasks are rooted in commonly used paradigms for studying intuitive physics (Figures 2A–2C), while others were added to increase the diversity of the scene and task structure (Figures 2D and 2E). In this way, we obtained a set of tasks whose key commonality was a demand for physical inference while they varied in the specific information necessary for the inferences. For example, the weights of individual blocks or discs were irrelevant to the judgments in the Toppling Towers and Bouncing Discs tasks, weight was the inferred property in the Bowling Balls and Weightlifting tasks, and the weight of the incoming ball was critical to the outcome in the Ramp Knock-Off task, along with the ball’s speed and position and the shape of the object it would collide with. While the diversity of scenarios inevitably contributed to individual differences in performance that were *not* related to intuitive physics and not shared across tasks (e.g., participants might vary in the precision of their visual motion estimates in tasks, which would affect some tasks more than others), it allowed for a more stringent test of whether competency in physical inference itself generalized across tasks. If people possess a flexible set of mental resources for intuitive physics, then an individual’s performance on one task should predict their performance on the others despite the myriad differences in task contents.

The split-half plots in Figure 2 show that individual differences in performance were highly reliable for all tasks in a sample of 71 online participants (correlations across independent split halves of trials: z = 0.59, p < 0.001 for Toppling Towers; z = 0.78, p < 0.001 for Bouncing Discs; z = 2.03, p < 0.001 for Bowling Balls; z = 0.87, p < 0.001 for Weightlifting; z = 0.69, p < 0.001 for Ramp Knock-Off). The split half correlation of subjects’ average physics ability across tasks was z = 1.42, p < 0.001. As noted above, this strong within-task reliability was crucial for testing our hypothesis; without it, there would be no basis for interpreting correlations (or lack thereof) in performance between tasks.

### Experiment 1: Testing for a general underlying intuitive physics resource

After establishing that each task in the battery captured reliable individual differences in performance, we turned our focus to the relationship of performance across tasks. Table 1 reports the pairwise correlations among tasks (These pairwise correlations are reported here as descriptive statistics, but the individual correlations were not the focus of our hypothesis testing. We anticipated that a substantial portion of the reliable within-task variance would not be shared across tasks because of the tasks’ considerable differences in contents and structure. Our subsequent analysis that isolated the common variance among tasks provided a more direct test of evidence for an underlying intuitive physics resource. The Bowling Balls task was the least correlated with the other tasks; as described below, this lower correlation is likely due to a well-known bias in participants' judgments when inferring mass from observed collisions. The magnitude of this bias varies across individuals independently of the precision of their mass discrimination, and the bias arises from perceptual processes rather than the physical inference itself.^9^^,^^10^ Nonetheless, the Bowling Balls task makes a valuable contribution to the overall set, providing a case in which participants must infer a latent physical property rather than predicting upcoming dynamics.). These positive pairwise correlations hint at the possibility of a common source of variance across tasks, but the relationships between various pairs of tasks could be driven by different sources of variance. To assess the shared variance within the task battery as a whole, we used a factor analysis to extract an underlying factor that could account for common variance shared across all tasks. Our approach here differed somewhat from other common applications of factor analysis and was aimed at addressing the specific question at the heart of this study – whether there exists a common resource driving individual differences in performance in all tasks. We had no *a priori* expectation that an underlying factor reflecting a general intuitive physics resource would explain all or even most of the variance in each individual task. In fact, based on theoretical grounds we anticipated the opposite – our tasks (and indeed, most every task used to study physical inference) draw on many facets of perception and cognition in addition to intuitive physics. For example, each of our tasks draws to some extent on visual attention, spatial reasoning, and working memory, to name a few. While these present a possible challenge to the specificity of any putative intuitive physics resource that must be addressed (as we do in Experiment 2 below), they also led us to expect that each task would have its own unique variance in addition to any common factor shared with other tasks.

**Table 1:** Correlations among intuitive physics tasks

	Toppling Towers	Bouncing Discs Task	Bowling Ball Task	Weightlifting Task	Ramp Knock Off Task
Toppling Towers	0.586 [0.40, 0.78]	0.568 [0.35, 0.77]	0.275 [0.053, 0.53]	0.580 [0.34, 0.82]	0.397 [0.14, 0.68]
Bouncing Discs		0.780 [0.54, 0.98]	0.386 [0.15, 0.66]	0.604 [0.42, 0.80]	0.344 [0.11, 0.59]
Bowling Balls			2.03 [1.55, 2.34]	0.271 [0.030, 0.60]	0.222 [0.026, 0.44]
Weightlifting				0.871 [0.68, 1.06]	0.537 [0.31, 0.82]
Ramp Knock- off					0.695 [0.42, 0.92]

Note: Gray background cells are within-subject split-half correlations. All other cells are complete subject pool correlations between tasks.

Table values are Fisher z scores of Pearson correlation coefficients. 95% confidence intervals are in brackets. Confidence intervals were computed using a bootstrapped distribution of 10,000 samples. Gray background cells are within-subject split half correlations. All other cells are complete subject pool correlations between tasks.

**Figure 3:**
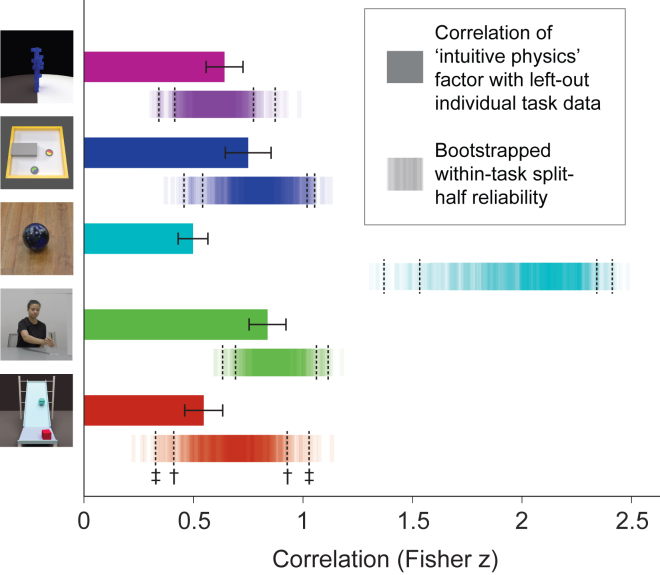
Variance in each task accounted for by a general ‘intuitive physics’ factor Each solid bar shows the correlation between the primary factor resulting from a factor analysis on half of participants’ data from all tasks and the independent, left-out data from each specific task. The error bars show one standard deviation of the bootstrapped distribution after repeating this procedure with 10,000 random splits. Shaded regions show the within-task split-half reliability for comparison, computed across 10,000 random splits. 95^th^ and 99^th^ percentiles of the bootstrapped split half reliability are indicated by † and ‡, respectively. The ‘intuitive physics’ factor accounted for a large portion of the variance in each task relative to the respective task’s split half reliability (R^2^ of mean factor correlation with task performance/R^2^ of mean split half correlation for each task: 1.14 for Toppling Towers, 0.95 for Bouncing Discs, 0.23 for Bowling Balls, 0.96 for Weightlifting, and 0.70 for Ramp Knock-Off).

We conducted a factor analysis using the collected data from the five tasks and took the first factor as a candidate index of general intuitive physics abilities. We then used a bootstrapped cross-validation procedure to test the degree to which this factor captured variance in independent left-out data from each task (see STAR Methods). Briefly, on each of 10,000 bootstrap iterations, we randomly divided each participant’s data from each task into two independent halves. We used one-half of the data to compute the general ‘intuitive physics’ factor, and then computed the correlation between that factor and each task’s left-out data. The results of this analysis are shown in Figure 3. The mean correlations between the ‘intuitive physics’ factor and the independent data from each individual task were as follows: Toppling Towers, z = 0.64, p <0 .001, R^2^ = 0.32; Bouncing Discs, z = 0.75, p < 0.001, R^2^ = 0.40; Weightlifting, z = 0.84, p <0 .001, R^2^ = 0.47; Ramp Knock-Off, z = 0.55, p <0 .001, R^2^ = 0.25; and Bowling Balls, z = 0.50, p < 0.001, R^2^ = 0.21. The accompanying shaded regions show the split-half reliability of each task for comparison. The ‘intuitive physics’ factor accounted for a substantial and significant portion of the variance in every task, and strikingly, with the exception of the Bowling Balls task, the variance captured by the single factor was not statistically different from the variance captured by the left-out data from the task itself. In other words, in most cases the ‘intuitive physics’ factor captured as much variance as it could *possibly* capture given the degree of noise in the data for each task. The Bowling Balls task is the exception – a single factor from the factor analysis captured significant variance in the Bowling Balls task but fell short of accounting for all explainable variance across individuals. This was not unexpected, however – there is a well-known systematic bias in judgments of relative mass from observed collisions, with people consistently overestimating the mass of the incoming ball.^10^^,^^11^^,^^12^^,^^13^ In our own recent work, we found that this bias contributes an independent source of reliable variance to individual differences in performance, and it arises from perceptual processes rather than the physical inference itself;^9^ see also.^10^^,^^14^ (The perceptual processes refer to visual estimates of physical variables such as speed, which serve as inputs to an intuitive physics system. Noise in these perceptual estimates can give rise to errors in the mass inference judgments, even if the intuitive physics system implements a veridical model of physical laws.). The unexplained variance in the Bowling Balls task here likely reflects individual biases that are specific to that task.

In sum, our findings in Experiment 1 revealed an underlying source of individual differences in intuitive physics performance across a variety of tasks, and we propose that these reliable individual differences arise from a mental resource for reasoning about the physical contents and dynamics of the world. Note that our finding of reliable individual differences does not imply that our lowest-performing participants are inept at intuitive physics. On the contrary, most everyone is an “expert” in physical reasoning and engages in it continually to interact with the world. Our tasks here are calibrated to zoom in on the dynamic range of performance differences across individuals, allowing us to leverage individual differences to test our hypotheses.

While the results of Experiment 1 point to a common mental resource contributing to performance on all tasks, the findings do not yet pinpoint the resource as being *specific* to intuitive physics. As noted above, other facets of cognition likely contribute to varying degrees to all tasks. For example, having strong mental rotation and spatial working memory abilities would likely improve a participant’s performance across all tasks to some degree. In Experiment 2, we tested whether intuitive physics abilities could be separated from the contributions of these other cognitive domains. In the process, we constructed a condensed, efficient version of our task battery that can be employed in myriad experimental contexts going forward.

### Experiment 2: Testing the specificity of intuitive physics resources

Our goal in Experiment 2 was to test the specificity of our intuitive physics measure by simultaneously measuring individual differences in intuitive physics performance and performance in other domains that might be alternative explanations. However, the task battery in Experiment 1 typically took almost 90 min to complete, which pushed the limits of the quantity of data we could collect within each individual participant while maintaining data quality; adding additional tasks would not be practical. To facilitate comparison of intuitive physics performance with other cognitive domains of interest, we sought to reduce the number of trials in the task battery while still capturing as much of the reliable individual differences as possible. We had a second motivation for reducing the battery size as well. To facilitate future work studying intuitive physics performance in patient populations, aging, over the course of training, etc., we wanted to provide a reliable measurement tool that could be conducted in a reasonable amount of time. For the sake of the present study, we prioritized capturing as much reliable variance from the full set as possible but aimed for the shortest trial set possible without sacrificing reliability.

**Figure 4:**
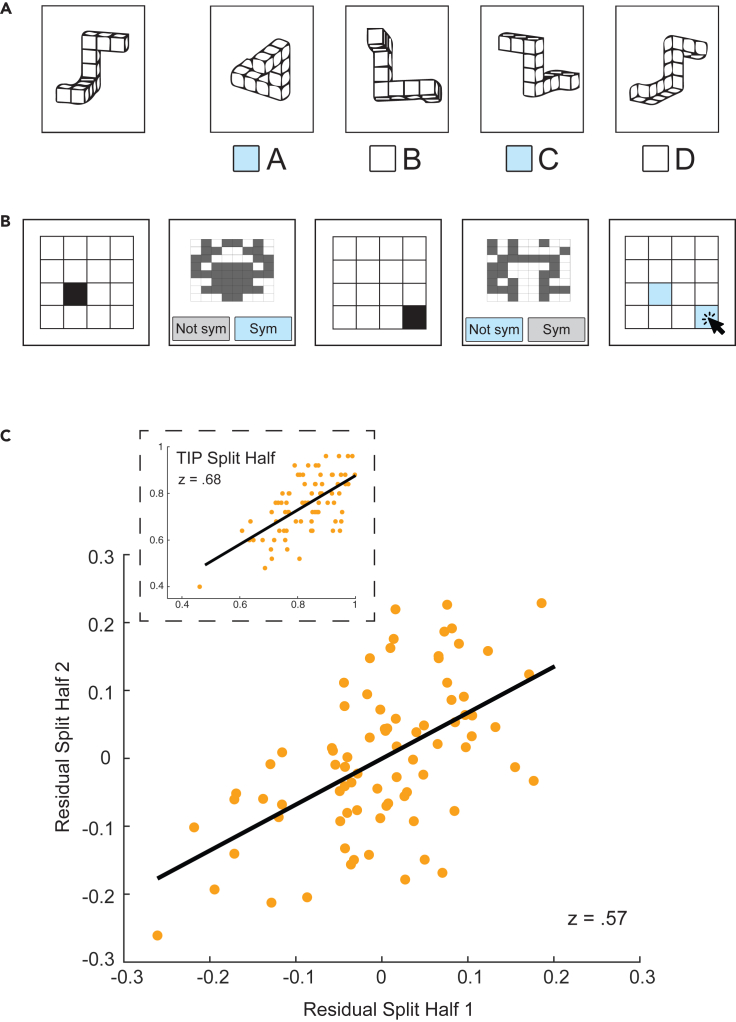
Experiment 2 additional tasks and results (A) The Mental Rotation Task (MRT).^15^^,^^16^^,^^17^ In each test item, participants are shown an image of a 3-dimensional shape (left) and asked to select the two shapes on the right that correspond to rotated versions of the shape on the left. The foil shapes (incorrect options) have been reflected about one axis and do not correspond to any possible rotation of the shape on the left. (B) Spatial Working Memory task. On each trial, participants were shown a 4x4 grid with a single black square filled in and were told to keep the position of the black square in memory. Before seeing the position of another black square, participants performed an intermediate task that consisted of a series of symmetry judgements on partially filled-in 8x8 grids. Participants were required to keep anywhere from 3 to 6 positions of black squares in memory before responding with their locations by clicking in an empty grid. (C) After regressing out the variance in intuitive physics performance (assessed with the shortened task battery; TIP) captured by the Mental Rotation and Spatial Working Memory tasks, the remaining variance still showed reliable individual differences, reflecting an independent contribution from physical reasoning not related to spatial cognition or working memory (z = 0.57, p < 0.001, N = 80 for the split-half correlation of the residuals after regressing out MRT and Spatial Working Memory). The inset plot shows the split-half correlation of the full (non-residualized) TIP scores for comparison.

To reduce the number of trials in the task battery, we used cross validated subsampling to find the trials that robustly captured the variance in the full battery (see STAR Methods). With a sub selection of 50 trials, 10 from each task, we were able to explain 92% of the variance in the full battery while shrinking the total time to complete the tasks to around 20 min. Compared to the full physics battery, our shortened version, which we are calling the Test of Intuitive Physics, or TIP, provides an almost equivalent measure of someone’s intuitive physics ability in a fraction of the time. The brevity of the TIP allowed us to test the specificity of our intuitive physics measure against an individual’s spatial reasoning and working memory ability. To do this, we ran the TIP alongside the Mental Rotation Task^15^^,^^16^^,^^17^ and a spatial working memory task that resembled complex span paradigms.^18^^,^^19^ In a sample of 80 online participants, performance on the TIP showed a positive split half correlation (z = 0.68, p < 0.001) indicating that our shortened battery still reliably captured individual differences in performance. The within-task correlation for the TIP was significantly stronger than the correlation between a split of the TIP and the Mental Rotation task (z = 0.39; p = 0.0062 based on a two-tailed bootstrapped test for the difference between correlations) as well as with the working memory task (z = −0.18; p < 0.0001, bootstrapped test). Note that we expected to observe some degree of positive correlation between the Mental Rotation task and TIP performance given the spatial aspects inherent in each intuitive physics task; see discussion. In a formal test of whether TIP performance could be fully explained by individual differences in spatial cognition and working memory, we ran two separate regression models on the split halves of TIP data with performances on the Mental Rotation task and the working memory task as predictors. These models explained 22% and 15% respectively of the variance in each split half of TIP performance. After regressing out the variance captured by the Mental Rotation task and the working memory task for each subject, the TIP split half remained significant (z = 0.57, p < 0.001) (Figure 4C). The positive split half relationship in the residuals suggested that the meaningful variation in the TIP was largely separable from spatial reasoning and working memory.

Performance on working memory tasks is highly predictive of fluid intelligence,^20^^,^^21^ so individual differences in performance on the working memory task in Experiment 2 almost certainly reflected individual differences in fluid intelligence as well. The findings here help to rule out the possibility that fluid intelligence was a primary driver of shared variance among the tasks in our set (to the exclusion of a separate resource for intuitive physics) – otherwise, we would not expect to observe such robust remaining variance in the residuals from the TIP after regressing out the contribution of the working memory measure.

### Experiment 3: Using the TIP measure to predict performance on a novel intuitive physics task

**Figure 5:**
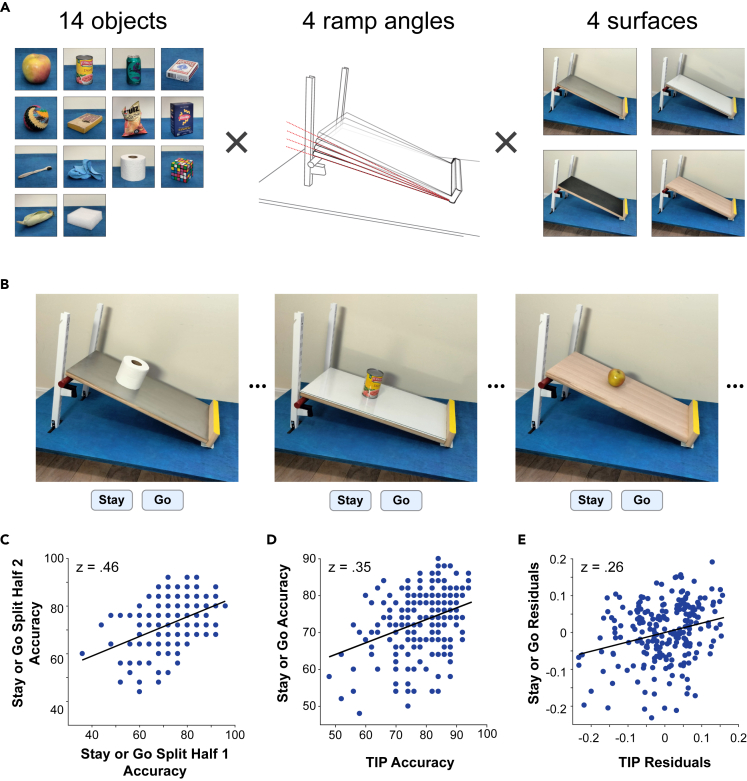
Experiment 3 Stay or Go Task and results (A) The Stay or Go Task. Left-Standalone images of the household objects used in the stimuli. Middle- The ramp varied in angle (12.02°, 14.94°, 17.85° or 20.77°). Right- The surface of the ramp was covered with one of four different materials (metal, glass, rubber, or wood). (B) Example stimuli. On each trial, participants saw one ramp/object configuration as pictured in the examples and were instructed to indicate whether the object would STAY in its current position when released or GO down the ramp (via sliding or rolling). (C) Split-half reliability of individual differences in Stay or Go task performance (scatterplot shows a representative split; z = 0.46, p < 0.0001). (D) TIP performance significantly predicted individual differences in performance on the Stay or Go task (z = 0.35, p < 0.0001). (E) Even after regressing out the variance in the TIP and the Stay or Go task that was captured by individual differences in performance on the Mental Rotations Test and Cambridge Face Memory Task, TIP performance significantly predicted Stay or Go performance (scatterplot shows residuals from the regression analysis; z = 0.26, p < 0.0001).

While Experiments 1 and 2 showed that performance on a heterogeneous set of intuitive physics tasks can be captured by a common underlying factor that varies across individuals (which is not simply an index of spatial and/or working memory abilities), the findings so far leave open the question of how well that factor can predict performance in a novel scenario that was not used in the construction of the measure. A key advantage of a mental resource for intuitive physics would lie in its flexibility – the ability to accommodate the wide variety of scenarios that we encounter in daily life. In Experiment 3, we sought to test the generalizability of the mental resource revealed by the prior experiments, using the TIP measure to predict performance on a novel task with different demands than the tasks in the initial battery. We constructed the Stay or Go Task (Figure 5) specifically for this purpose, placing a greater emphasis on surface friction and object geometry than the previous tasks and lesser demand on tracking physical dynamics. The stimuli consisted of photographs of recognizable objects positioned on a ramp, which varied in its steepness and surface material (Figure 5A; see STAR Methods). Participants were tasked with judging whether the object would remain stationary under the effects of gravity or would move down the ramp either by rolling or sliding. The ground truth for the experiment came from real-world outcomes when each trial configuration was photographed; all trials that were ultimately used in the experiment had clear-cut real-world outcomes, and objects that would slide or roll down the ramp were affixed with a hidden piece of tape to keep them stationary and positioned naturally for the photographs (see STAR Methods). Figure 5B shows three example trials from the set. We initially photographed all combinations of the fourteen objects, four ramp angles, and four surface materials for a total of 224 stimuli. Similarly, to the tasks in Experiments 1 and 2, we conducted pilot testing to identify the trials that most reliably captured individual differences in performance, validating the trial selection in independent left-out data. The final resulting task consisted of 50 trials and included all ramp angles and surface materials.

In the main experiment, we presented online participants with the Stay or Go Task as described above, along with the TIP measure established in Experiment 2, the Mental Rotations Test, and the Cambridge Face Memory Test.^22^ The Cambridge Face Memory Test is a widely used measure of face processing abilities; we included the task as an additional measure of cognitive differences outside the domain of intuitive physics to help interrogate the nature of the relationship between performance on the TIP and the Stay or Go task. We collected data from 239 online participants, 219 of whom were included in the final analysis after excluding participants for whom we did not acquire complete data for all tasks. In line with our piloting of the Stay or Go Task, a split-half analysis revealed reliable individual differences in performance (Figure 5C; split-half correlation of z = 0.46; p < 0.0001). The other tasks also captured reliable individual differences in performance as measured by split-half correlations (z = 0.53 for the TIP, z = 1.23 for the Mental Rotations Test, and z = 1.14 for the Cambridge Face Memory Test; all p < 0.0001). After verifying the reliability of the individual measures, we turned to testing whether performance on the TIP could be used to predict performance on the new Stay or Go task. Figure 5D shows the relationship between individual differences in performance on the two tasks; Stay or Go performance correlated significantly with the general measure of intuitive performance provided by the TIP (z = 0.35; p < 0.0001). Similar to the approach in Experiment 2, we then used performance on the two additional non-physics tasks to aid in evaluating whether the relationship between the TIP and the Stay or Go Task was mediated by other cognitive abilities that might contribute to both tasks. The Mental Rotations Test was included to account for the contribution of spatial abilities, and the Cambridge Face Memory Test was included to capture other general cognitive/performance aspects that vary reliably across individuals (for example, perceptual abilities or level of effort devoted to the tasks). Performance on each of the non-physics tasks correlated with performance on the Stay or Go Task (to a lesser degree than the TIP but still significantly; z = 0.21, p = 0.002 for Mental Rotations; z = 0.26, p = 0.0004 for Face Memory). We expected to observe these positive correlations because the Stay or Go task necessarily draws on multiple facets of cognition – for example, assessing the objects’ 3-dimensional shapes is important for determining the outcomes of the scenarios and draws on perceptual and spatial abilities. The key question of interest is whether the abilities these tasks measure are responsible for mediating the relationship between performance on the TIP and the Stay or Go Task. We assessed this possibility using a regression analysis as in Experiment 2, partialing out the variance in Stay or Go and TIP scores accounted for by performance on the Mental Rotations Test and the Cambridge Face Memory Test. Figure 5E shows the correlation in the residuals from the regression analysis – after removing the variance that could be accounted for by the non-physics tasks, TIP residual scores still significantly predicted performance on the Stay or Go task (z = 0.26, p < 0.0001). These findings establish that the mental resource uncovered in Experiments 1 and 2 generalizes to predict performance in a novel physical scenario that differs in its contents and demands.

Of course, the tests here do not exhaustively evaluate all the possible sources of shared variance among the tasks in our set – testing the distinctness of the intuitive physics system from other facets of cognition will be an ongoing endeavor (see discussion). However, the marked differences in scene content and inference demands among our tasks help to concentrate their commonality on the demand for physical reasoning as much as possible, while our control tasks in Experiments 2 and 3 help to rule out the most likely alternative explanations. It is also unlikely that individual differences in the strength of visual imagery underlie the correlated performance across intuitive physics tasks. Previous work from our lab has examined the relationship between the vividness of visual imagery and performance on intuitive physics tasks, showing that there is essentially no correlation between the two.^23^ While this result may at first seem counterintuitive from the viewpoint that people might form their physical predictions by seeing a “movie” of the dynamics play out in mental imagery, it accords with brain imaging findings situating intuitive physics processes within the dorsal processing stream and not necessarily closely linked to the phenomenology of visual experience.^7^^,^^8^

## Discussion

We measured performance on a collection of physical inference tasks, each one calibrated to capture reliable individual differences and consisting of a unique combination of scene contents and task demands. Despite considerable differences across tasks in the nature of the required inference and the relevant physical variables, we found that individual differences in performance were tightly correlated among the tasks, pointing to a common mental resource contributing to the physical inferences in all scenarios (Expt. 1). The relationship among intuitive physics task could not be accounted for by individual differences in spatial abilities or working memory (Expt. 2), and it predicted performance on a novel physical inference task that emphasized different scene contents than the original tasks (Expt. 3). Collectively, these findings provide evidence for a dedicated intuitive physics system in the mind with sufficient flexibility to accommodate a wide range of scenarios.

Our results accord with a number of existing findings from the areas of human development, functional brain imaging, and computational modeling, all pointing toward the possibility of a domain-specific intuitive physics system.

1) An extensive body of developmental work has shown that physical knowledge relating to the concepts such as support, solidity, and continuity emerges in highly systematic fashion during the first years of life.^24^ For example, in a stereotyped developmental time course, infants come to expect a suspended object will fall unless it is in contact with another object, then expect that it will fall unless it is *on top* of the other object, and then expect that it will fall unless its approximate center is positioned over the supporting object.^25^ These physical expectations quickly improve in sophistication and breadth; to give one example, by just five months of age infants have distinct expectations about liquids and solids and are attuned to a variety of expected liquid behaviors.^26^^,^^27^ By eleven months old, infants use their surprisal about unexpected physical behaviors to guide their exploration-based learning via hypothesis testing.^28^ The systematicity with which infants acquire expectations about physical principles and use them to guide their behavior is suggestive of an innate core system for physical reasoning that scaffolds the emergence of intuitive physics abilities.
2) Recent neuroimaging work has uncovered a network of brain regions that are recruited for physical inference over and above other difficulty-matched tasks.^29^^,^^30^ These regions might constitute a “physics engine” in the brain, and subsequent findings have bolstered this idea. The same regions encode information about objects’ masses in a scenario-invariant fashion,^31^ and they are precisely targeted by functional connections from the dorsal anterior cingulate cortex, a key structure for selecting scene contents based on goal-driven action planning.^32^ The reliable engagement of this brain network during physical inference tasks hints at the prospect of a dedicated mental resource for intuitive physics.
3) A third line of evidence comes from recent computational work showing that people’s physical judgments are well-modeled by an approximate Newtonian model that applies a basic set of physical laws to noisy perceptual inputs to simulate what will happen next.^1^^,^^33^^,^^34^^,^^35^ Even in cases where people’s judgments appear to be at odds with real-world physical behaviors, such models have been successful in accounting for these apparent misconceptions by allowing for imperfect or incomplete inputs to the mental simulation model.^14^^,^^36^^,^^37^ Thus, a compact set of physical principles can account for human performance across a range of intuitive physics tasks.

Perhaps the most immediate question following from the current work is what *kinds of mechanisms* make up the mental resource for intuitive physics. Our findings argue for the existence of such a resource, but its constituent algorithms could take a number of possible forms. One form, touched on in the Introduction, is mental simulation akin to a video game physics engine.^1^^,^^35^ A simulation-based intuitive physics system would predict future states of the world by “playing forward” the dynamics of a scene based on a description of the relevant variables governing its behavior. Simulation-based solutions would not necessarily be perfect – just as video game engines make simplifications to rapidly approximate physical behavior, a simulation-based mental model might do the same, especially given that much of our physical reasoning must happen rapidly enough to guide online actions.^7^^,^^8^^,^^38^ People’s predictions on a variety of tasks do indeed match those of noisy simulations^1^^,^^14^^,^^39^^,^^40^^,^^41^^,^^42^ and simplifications introduced to such simulations can lead to patterns of errors that match human observers’ errors.^36^ Still, mental stimulation is not the only possible way of implementing a flexible intuitive physics system. Rule-based reasoning could potentially achieve comparable generality, *so long as a core ruleset pertains to general physical behaviors that are observed across a wide variety of scenarios.* While the scenario-specific rules illustrated in Figure 1A fail to generalize, rules such as “gravity pulls objects downward” or “a cube will slide rather than roll” could be applied to reason about many everyday scenarios. During development, the first hints of physical understanding appear to be rule-based.^25^ While some rules are first acquired in a scenario-specific fashion, they pertain to general physical behavior and are eventually unified into a more abstract concept that applies across scenarios. Adult physical reasoning could be a highly elaborated and refined version of the same rule-based system. Rule-based and simulation-based intuitive physics systems are not mutually exclusive; it may be that people employ both, trading off between approaches depending on which is most appropriate for the current goals, or integrating information from the two.^43^ The critical distinction between this kind of rule-based system and the one highlighted in Figure 1A is that these rules *implement Newtonian principles*, whereas those in Figure 1A leverage stimulus-outcome contingencies that need not have anything to do with the laws of physics. On top of the general physical prediction mechanisms discussed here, it may be that extensive experience with a particular scenario builds a repertoire of stimulus-outcome contingencies that can augment the predictions of the general system, for example in expert billiards players determining ricochet angles.^3^ In sum, the mechanisms of intuitive physics may be diverse, but our present work suggests that a central component of the system applies general physical principles to understand a variety of scenarios.

How does a dedicated resource for intuitive physics fit into the broader architecture of the mind? Whether rule based, simulation engine, or other mechanism, the intuitive physics system certainly does not operate in isolation. For example, in nearly all of the everyday scenarios that demand physical reasoning, and certainly in all of the tasks in the present study, people must draw on their spatial skills to some degree to represent the layout of the scene and the geometry of the objects in it. A host of perceptual processes contribute to forming accurate spatial representations, and to inferring surface textures that are important for physical inference (see^44^ for evidence of contribution of visual cortex to mental simulation of physical behaviors). All of these processes collectively contribute to our successes and failures in everyday physical reasoning and are bound to be intimately linked with the intuitive physics system in the brain. Indeed, prior work from our lab has shown a strong correlation between subjects’ spatial abilities and their performance on intuitive physics tasks.^9^^,^^45^ The fact that intuitive physics is *separable* from these other facets of cognition should not be taken to mean that it operates independently of them. The intuitive physics system might also be tightly linked with action planning and tool representation; the primary computational goals of these processes might actually be the same – to support effective and efficient first-person interaction with the world.^7^^,^^8^ At the same time, intuitive physics may truly operate independently from some facets of the mind. There is evidence from work with patient populations^46^ and neuroimaging studies in typical adults^30^^,^^32^ that social cognition may be one such case – physical reasoning skills and social skills appear to vary independently across individuals and draw on largely non-overlapping brain networks. Overall, the work to situate intuitive physics within the broader landscape of cognition is just getting underway but work thus far hints at it being a nexus of sensorimotor function.

Alongside the direct scientific implications of this study for understanding the intuitive physics system in the mind, our study also contributes a valuable resource for other researchers who wish to measure intuitive physics abilities: the TIP. We were excited to find that a task battery of dramatically reduced length was able to capture the vast majority of the reliable variance from the full task set used in Experiment 1. The TIP provides a sensitive and reliable measure of general intuitive physics ability in a time frame that is reasonable for use in many clinical populations, and it opens the door to exploring the relationship between intuitive physics and a host of other cognitive abilities. It can also be employed longitudinally to investigate the effects of both training and aging. For example, does athletic training lead to general improvements in physical inference abilities, or are the training effects restricted to the athlete’s specific sport? Our hope is that the TIP proves to be a valuable research tool in a variety of contexts.

### Limitations of the study

Two limitations of the present study relate to design decisions made in the interest of keeping the work focused and tractable. The first limitation is the strength of the claim we can make about the *specificity* of the underlying mental resource to intuitive physics. As discussed in the opening of the paper, the existence of domain-specific mental processes has itself been an enduring issue of contention. The challenge in arguing that a component of the mind or an area of the brain performs a specific set of computations to the exclusion of all others is that demonstrating specificity with certainty would require testing and rejecting *everything else* that it might plausibly be involved in. Short of devoting unlimited time and resources, specificity arguments must always grapple with the possibility that some untested stimulus or task would engage the process of interest in an unexpected way. This is certainly a possibility that our present work must grapple with, and we do not wish to overstate the case that can be made from this single study by itself. We aimed to test the prime suspects – other cognitive abilities that pose the most plausible alternatives as a unitary driver of performance across our tasks. More studies are needed to strengthen the argument for specificity, but importantly, even the finding that this ‘intuitive physics’ resource is involved in other non-physics tasks as well would not undercut what our work has uncovered about how physical inference is achieved in the mind. That is to say, the story in Figure 1 would not fundamentally change if the ‘general physics system’ in Figure 1B supported, say, tactile pattern learning as well.

A second limitation of our present work similarly relates to a design decision that we made with careful consideration to keep the scope of the work focused. Our tasks here focus on rigid-body interactions and do not include examples of liquids, gases, cloth, soft-body objects, and other materials. There were two main reasons for focusing on rigid bodies in this study. One consideration is that we aimed to ground the present work in the existing literature on intuitive physics. While the study of people’s ability to reason about liquids and soft bodies has received increased attention as of late, demonstrating some cases in which people are successful^26^^,^^39^^,^^47^^,^^48^^,^^49^^,^^50^ and others in which people have difficulty,^51^^,^^52^ the vast majority of the research on intuitive physics is still focused on rigid body interactions.

A second and more important consideration motivating our focus on rigid bodies is the need for the appropriate kind of heterogeneity among tasks for testing transfer of performance as depicted in Figure 1. That is, we needed a set of tasks for which a common model of physical dynamics and a common set of physical variables *could potentially* be employed to understand each scenario despite the differences in scene contents. A drawback of including continuously deforming scene contents such as liquids and soft bodies in our tasks would have been that there is reason to suspect *a priori* that reasoning about such cases would require a different mental model. They are described by different equations in the study of physics, and a host of additional variables are critical for modeling their behavior (e.g., viscosity, stiffness, plasticity). Introducing differences of this scale into our task set from the outset would have made it more difficult to evaluate the apparent similarity of the mental resources supporting the rigid-body subset of the tasks. To be clear, our view is that ultimately, a complete model of intuitive physics must include all the varieties of physical content that we interact with in daily life; here, we focused the scope of the work in order to make an important step in that direction.

## STAR★Methods

### Key resources table

**Table:** 

REAGENT or RESOURCE	SOURCE	IDENTIFIER
**Software and algorithms**		

Blender 3-D modeling software	Blender	

### Resource availability

#### Lead contact

Further information and requests for resources should be directed to and will be fulfilled by the lead contact, Alex Mitko (amitko1@jh.edu).

#### Material availability

The full stimulus set from this study is available at https://www.dynamicperceptionlab.com/resources.

#### Data and code availability

• All data reported in this paper, will be shared by the lead contact upon request.
• All original code will be shared by the lead contact upon request.
• Any additional information required to reanalyze the data reported in this paper is available from the lead contact upon request.
• This study was not preregistered.

### Experimental model and study participant details

#### Experiment 1 participants

All participants were recruited on Amazon’s Mechanical Turk (mTurk) and were required to be 18-35 years of age. All participants were also required to complete the study from within the United States. Information on sex, gender, and ethnicity was not collected for the 3 experiments. The order in which the tasks were presented was randomized for each subject. A total of 155 mTurk workers completed the tasks, 44 of whom were excluded prior to any further analysis for the following reasons: 31 participants attempted to complete multiple tasks at the same time, 10 participants were not located in the United States, and 3 participants experienced internet glitches that resulted in missing or incomplete save files. We also established an additional exclusion criterion that was applied after preliminary analysis of raw accuracy scores: a participant was excluded from further analysis if their performance on two or more of the tasks was below chance level (50%). We established this criterion as a principled way to exclude participants who did not understand the task instructions or did not make a good faith effort to complete the tasks (with online data collection, it is common to encounter some instances of tasks being completed by automated bots that make random responses). Because we did not wish to exclude even the lowest-performing participants who made an effort to complete the tasks, each task included “easy” trials for which nearly all participants gave correct responses in our piloting. Applying this performance cutoff resulted in the removal of an additional 40 participants from subsequent analyses, leaving a total of 71 participants. Two participants experienced malfunctions in loading more than 25% of the videos in the Weightlifting task and were therefore excluded from the Weightlifting split half analyses and the factor analysis. Participants were paid US$10 to complete the study and provided anonymous informed consent in accordance with Johns Hopkins University Institutional Review Board (IRB) protocols.

#### Experiment 2 participants

All participants were again recruited on Amazon’s Mechanical Turk and the same exclusion criteria were applied. A total of 249 mTurk workers completed the tasks, 47 of whom were excluded before analysis for the following reasons: 12 participants attempted to complete multiple tasks at the same time, 8 participants were not located in the United States, 12 participants experienced internet glitches that resulted in failed save files, 11 participants were missing trials (which were crucial to an already shortened battery), and 4 participants gave the same response on all trials. An additional 122 participants were excluded based on a performance cutoff on the working memory task: we excluded participants who scored a 0 out of 12 on the grid location trials and we excluded those who scored under 85% on the symmetry judgements (these judgments were trivially easy and included, in part, to identify participants who did not understand the task or did not make a good faith effort to complete it). This left a total of 80 participants for analysis. Participants were paid US$7 to complete the study and provided anonymous informed consent in accordance with Johns Hopkins University Institutional Review Board (IRB) protocols.

#### Experiment 3 participants

All participants were recruited on Prolific and the same exclusion criteria as Experiment 2 were applied. A total of 239 participants completed the tasks, 20 of whom were excluded before analysis due to restarting tasks and/or having missing trials due to internet issues. Participants were paid US$8 to complete the study and provided anonymous informed consent in accordance with Johns Hopkins University Institutional Review Board (IRB) protocols.

### Method details

#### Experiment 1: Intuitive physics battery

The intuitive physics battery consisted of 5 tasks: Toppling Towers, Bouncing Discs, Bowling Balls, Ramp Knock-off, and Weightlifting. As described in the results, these tasks were designed to vary not only in their scene contents, but also in the type of physical judgement – some required inferring a latent variable (mass) while others required a prediction of how motion trajectories would unfold. The five tasks were presented one at a time in a randomized order. Example stimuli for each task are shown in Figure 2 and the complete stimuli set can be found on the lab’s website: http://www.dynamicperceptionlab.com/resources.

##### Toppling Towers

The Toppling Towers task was the same one used in Mitko and Fischer^45^ and based on previous studies with a similar design.^1^^,^^29^^,^^41^ Stimuli were made using Blender 3-D modeling software (http://www.blender.org). On each trial, participants were shown a 360-degree camera pan of an unstable block tower positioned at the center of a platform. Participants were informed that the tower was shown in a freeze frame of the moment before it would fall. The platform that each tower sat on was colored grey on one half and white on the other. On every trial after seeing the video of the tower, participants were tasked with indicating which side of the platform the majority of blocks would come to rest after the tower fell. Participants were presented with one practice trial that showed the 360-degree pan followed by the tower falling down, which allowed them to observe the blocks’ physical properties. On the remaining trials used for analysis, the toppling behavior was not shown, and the experiment proceeded to the next trial after a participant’s response. Participants were shown 48 different tower videos and used a mouse click on every trial to indicate on which side the majority blocks would come to rest. While the participants made their response, the last frame of each video remained on the screen. The towers consisted of 11, 13, 15, 17, 19, or 21 blocks (8 towers of each, 4 of which would fall to the gray side and 4 to the white side). The trials were presented in a randomized order for each participant.

##### Bouncing Discs

In the Bouncing Discs task participants saw two discs sliding around a square ‘arena’. One of the discs was half blue/half green and the other disc was half red/half yellow. After bouncing around the arena for 2 seconds, one of the discs became invisible for the next 2 seconds. Following this period of the one disc’s invisibility, it reappeared in the scene and the two discs continued bouncing for the last 2 seconds of the video. On every trial, participants made a response indicating whether the invisible disc reappeared with the correct position and velocity that would be expected if it continued to move within the arena while it was invisible. Participants made their responses by clicking a ‘Yes’ or ‘No’ button with their mouse. In half of the trials, the position of the invisible disc was offset to a nearby location and its velocity was randomly reassigned just prior to its reappearance. In the remaining half of the trials, the invisible disc reappeared in the correct position with the correct velocity, as though it had continued to interact within the scene while it was invisible. There were four ‘arenas’, each with a different barrier layout to add variation to the discs’ interactions. The color of the disc that became invisible was counterbalanced across trial types and arenas to encourage participants to track the behavior of both discs prior to one of them becoming invisible. A total of 64 stimuli were shown in a random order to each subject. Stimuli were made using Blender 3-D modeling software, and physical outcomes were assessed using simulations run in Blender’s built-in Bullet physics engine. Before testing, participants performed 3 practice trials in which they were given feedback on the accuracy of their responses and shown a second video depicting the scene dynamics with both discs visible for the full video.

##### Bowling balls

On every trial of the bowling ball mass interference task, participants saw a video of a collision between two bowling balls and were asked which bowling ball was the heavier of the two. The videos were filmed in the lab using real bowling balls, with an out-of-frame ramp launching the incoming ball at a consistent speed. The weights of the balls were 6, 8, 10, 12, 14, and 16 pounds and all had the same blue wavy surface pattern. Each collision consisted of a stationary ball contacted by a ball rolling in from the left side of the screen (incoming ball). The incoming ball was held at the top of a ramp and released to launch it into the video frame where it collided with the stationary ball. This procedure was completed twice for all 30 possible pairs of weights, totaling 60 stimuli. Each video ended shortly after one of the balls left the frame, which lasted approximately 3 seconds. Participants saw each stimulus video four times, totaling 240 trials. After seeing each video, participants selected a button labeled ‘Ball 1’ or ‘Ball 2’ to indicate which ball they thought was heavier, with Ball 1 always referring to the incoming ball. The question ‘Which ball is heavier?’ always remained on the screen. After making a selection, participants pressed another button to advance to the next trial. Participants were shown a single practice trial before the task began.

##### Weightlifting

On each trial of the weightlifting task, participants viewed two short video clips of an actor picking up an object, lifting it to touch an upper target, and placing it back down. The task was to determine in which video was the object heavier. The objects were visually identical but varied in weight. Twenty metal canisters were prepared that ranged in weight from 100 grams to 2,000 grams in increments of 100 grams. Each canister was filled with enough metal BBs to bring it up to its final weight. The BBs within each canister were enclosed in a plastic tube that allowed BBs to be centered horizontally and distributed evenly along the full height of the canister. Packing material around the BBs prevented any shifting of the internal weights when the canister was picked up or moved. Four members of the Johns Hopkins community (Lifters) were recruited for the creation of stimulus videos. Lifters were unaware of the hypotheses involved in this set of experiments. Lifters were filmed as they picked up each of the twenty canisters, lifted it to a predefined height, then lowered it and placed it on the table. During filming, the canisters were lifted in a randomized order that differed for each lifter. Each lifting event began with a canister resting on a foam start square on the table in front of the lifter. Lifters began with their hand flat on the table, and when cued by a signal light, reached out to pick up the weight and lifted it to a predetermined target height as indicated by a white board. Lifters then lowered the weight back to its starting square and replaced their hand in its starting position. All clips were approximately 6 seconds long. Videos from the ten weights were paired to make five unique weight combinations (100g and 2000g, 300g and 1800g, 500g and 1600g, 700g and 1400g, 900g and 1200g). Each pairing was shown twice for each Lifter, switching which clip was shown first. Each trial contained two videos of the same lifter. The two videos were separated by a black screen; the inter-stimulus interval (ISI) was 1.0 second. After the second clip finished playing, the video screen turned black again. Participants were prompted with the question “Which is heavier?” and clicked one of two radio buttons to indicate which video depicted the heavier object being lifted. This response was self-paced, and the task would only continue once participants made a selection and clicked the submit button. The task consisted of 40 trials with self-timed breaks after every 10 trials. No feedback was provided.

##### Ramp Knock-off

In the Ramp Knock-off task, participants were presented with still images of scenes in which a ball was positioned at the top of a ramp, ready to be released. In each scene, a second object was situated on a platform at the bottom of the ramp, and participants were tasked with judging whether the ball would knock the object below off the platform when it was released. The objects on the lower platform were simple geometric solids – a cube, cylinder, or pyramid. The masses of these objects were held constant across all trials (all had the same density, so the masses of different objects varied according to slight differences in their volumes). The mass of the ball at the top of the ramp varied across trials, and its mass was indicated with a label on its surface – “light” or “heavy”. The angle of the ramp also varied across trials, with three possible angles achieved by supporting the ramp with bars at varying heights. Ramp angle, ball weight, and the shape of the lower object varied independently of each other, and we used a generative algorithm to create a trial set that balanced the outcome of the collision (whether the lower object was knocked off the platform or not) with respect to each of these variables. The generative algorithm produced a large set of scenes by randomly positioning the ball and the second object below, and randomly assigning the ramp angle, ball weight, and shape of the lower object. The outcome of each scenario was simulated using Blender’s built-in Bullet physics engine, and a subset of the full family of generated scenarios was selected to balance the collision outcome for each of the relevant variables so that the object was knocked off 50% of the time. The final trial set contained 58 trials, which were presented in a random order to each participant. During the experiment, participants viewed a still image of each scene and were asked “Will the red object be knocked off?” Participants responded as soon as they had reached a decision by clicking one of two buttons labeled yes and no. No feedback was provided. Before starting the task, participants were shown three videos of ramp collision scenes that were not used in testing in order to observe the objects’ properties.

#### Experiment 2: Testing the TIP against spatial ability and working memory

##### Test of Intuitive Physics (TIP)

For the purposes of testing intuitive physics against other forms of cognition, we needed to shorten the length of our task battery while maintaining its capacity to capture reliable individual differences. To slim the battery down, we iterated a sub-sampling procedure that randomly drew 10 trials from each task and compared each subject’s mean performance on these 50 trials to their mean performance on the full physics battery. The sample with the highest correlation after 10,000 iterations was able to explain 92% of the variance in the full battery and thus became the Test of Intuitive Physics, or TIP. The instructions for each physics task in the TIP, as well as the practice trials included in the full battery, were shown to participants before starting each task. This shortened version takes about 20 minutes to complete.

##### Mental Rotation Task

The Mental Rotation task consisted of 24 questions that each showed a target stimulus of connected blocks.^15^^,^^16^^,^^17^ Participants were required to select two out of a possible four response options, two correct options that were rotated versions of the target stimulus and two incorrect options that were structurally different than the target stimulus. There were two correct responses for each question, so accuracy was determined by dividing the total number of correct responses by 48. All the questions were presented on the screen at the same time and there was no time limit for completing the task.

##### Working memory task

The working memory task was based on commonly used complex span tasks^18^^,^^19^ and was a slight variation of a working memory task used in our previous work.^45^ It consisted of performing two tasks in alternation, remembering the position of black squares on a grid, and determining whether a matrix was symmetrical. Each trial started by showing a 4x4 grid of squares, with a single black square filled in. After 1500 ms, the grid was taken off the screen and a new 8x8 grid appeared with some blocks colored gray and others colored white. Participants clicked one of the two buttons on screen to respond whether the colored in blocks made the grid horizontally symmetrical. Four symmetry judgements were presented in a row before the 4x4 grid returned with another single black square. This process repeated 3-6 times after which a blank 4x4 grid was shown and participants were instructed to click on the squares that had been blacked out since the previous sequence. Selected grid locations were highlighted in yellow, and participants were able to select and unselect positions before pressing a submit button. The order of the position selection was not emphasized to the participants, nor analyzed. A response was considered correct on the memory grid if all positions were correctly recalled. Accuracy on the in-between symmetry judgements was used for exclusion purposes. Participants completed three trials of each sequence length.

#### Experiment 3: Using the TIP measure to predict performance on a novel intuitive physics task

##### Stay or Go Task

In the Stay or Go Task, participants were required to determine whether a household item positioned on a ramp would slide or roll to the bottom when released. This task draws more heavily than the previous ones on participants’ ability to incorporate estimates of surface friction and complex object geometry into their judgments. On each trial, subjects saw a static image of a household item positioned on a ramp; they pressed one of two buttons on the screen, “Stay” or “Go”, to indicate whether the object would remain stationary due to friction or would travel down the ramp (either rolling or sliding) when released. The images were photographs of real-world configurations, taken from the same angle with each item placed in roughly the same location along the ramp. To obtain static images that did not contain motion blur or other cues to an object’s movement, the objects were stuck in place with tape that was not visible in the images. We ensured that the tape held the objects in a natural resting position by requiring that the taped objects exhibited no movement (e.g. a slight slide or forward tilt) when released. The stimuli varied in the object presented (14 household items; Figure 5A), the ramp angle (12.02°, 14.94°, 17.85° or 20.77°; Figure 5B), and the surface material of the ramp (wood, glass, metal, or rubber; Figure 5C). As with the previous tasks, we used an iterative piloting process to determine which 50 stimuli out the full set of 224 were most reliable in discriminating individual differences in performance. The final set of stimuli consisted of equal numbers of Stay and Go trials, and the other scene variables were roughly counterbalanced. During the main experiment, the stimuli were presented in a randomized order and no feedback was given. At the start of the experiment, a task instructions page showed images of all 14 items with labels, and participants were shown a movie of an object sliding down each of the four ramp materials to familiarize them with the setup (the objects in the movies were distinct from the 14 objects used in the main experiment). Performance on the task was computed as percent correct, with the ground truth answers given by the real-world behaviors of the objects recorded during the stimulus creation.

##### Cambridge Face Memory Task

This task was adapted from Duchaine and Nakayama^22^ for online testing. The task involves 3 testing phases: In the first phase, after being shown images of individual faces, participants must identify the previously seen faces among distractor faces. In the second phase, after reviewing the target faces for 20 seconds participants must identify novel images of the same individuals. In the final phase, after reviewing the set of faces for 20 seconds, participants must identify novel images of the previously seen individuals, this time with Gaussian noise added to the images. For further details see Duchaine and Nakayama.^22^

### Quantification and statistical analysis

#### Experiment 1

##### Split half analyses

To determine the reliability of each task we performed split half analyses. This involved randomly splitting the trials of each task and comparing subjects’ performance on the two independent halves of the trials. Using this method, a reliable task would result in a positive correlation between participants’ split halves. Note that this is a conservative estimate of reliability because it splits the full set of trials in half, whereas all trials were used in subsequent analyses comparing performance across tasks. To test for significance, we performed permutation tests, first conducting the random split half procedure 10,000 times and calculating the mean Fisher z score value to ensure that an unrepresentative split could not affect the outcome of the test. We used this distribution of Fisher z score values to calculate 95% confidence intervals. To test for the overall reliability of the battery, we first computed the mean performance on each task’s split half. Then, for each subject, we averaged each task’s split half performance to obtain a combined physics measure for each split half. We then computed the correlation of the combined physics measure across subject, between the two split halves. We then repeated this procedure, this time shuffling the subject correspondence between split halves in each iteration, and took the resulting distribution of correlation values as a null z score distribution. We then compared the mean z score value from the non-shuffled data to the null distribution obtained from the shuffling procedure to determine significance for the split half correlation.

##### Split half factor analysis and validation

To test whether a single underlying factor explained significant variance across all tasks, we conducted a split-half procedure in which a factor analysis was conducted on one half of the data and validated by testing the variance it explained in each task in the independent, left-out half of the data. In the validation step, we computed the correlation between the factor and the left-out data for each physics task to determine how well this single factor could explain variance in each task. We iterated this process 10,000 times and calculated the mean of the resulting correlations after applying a Fisher z-transform to allow for linear comparison of the correlations. For statistical testing, we created a null distribution by shuffling the participants so that the split halves for the factor correlation were no longer aligned, and then we compared the mean correlation to the null distribution.

#### Experiment 2

##### Design and analysis

With our shortened version of the physics battery, we examined the relationships between intuitive physics and spatial ability, as well as intuitive physics and working memory. We had online participants perform the TIP, the Mental Rotation task, and the spatial working memory task. The order of the tasks within the TIP was fixed in the following sequence, Bowling Balls, Bouncing Discs, Toppling Towers, Ramp Knock-off, and finally Weightlifting. We used a fixed task ordering within the TIP for the sake of consistency in its future application as a measure of intuitive physics abilities. The order in which the TIP, Mental Rotation task, and the working memory task were presented was randomized and participants were encouraged to take breaks in between tasks. To determine if our measure of intuitive physics was separable from spatial ability and working memory, we tested whether reliable individual differences remained after regressing out the variance captured by the Mental Rotation task and the working memory task. First, we randomly split each task within the TIP in two and calculated the mean physics performance on split halves of the TIP data. Then, we ran a linear regression on both halves predicting mean physics performance from Mental Rotation performance and working memory performance. A correlation of the residuals from these two regressions indicated the leftover variance in TIP performance that could not be explained by the Mental Rotation and working memory tasks. For statistical testing, this process was iterated 10,000 times and the mean correlation value was compared to a null distribution that was created by shuffling the split half alignment.

#### Experiment 3

##### Design and analysis

The design and analysis of the data from Experiment 3 were essentially identical to the approach in Experiment 2, applied to a different collection of tasks. Within a single session, each participant performed the Stay or Go task, the 5 tasks in the TIP, the Mental Rotations Test, and the Cambridge Face Memory Task.
